# Thromboxane A_2_ Receptor Stimulation Promotes Closure of the Rat Ductus Arteriosus through Enhancing Neointima Formation

**DOI:** 10.1371/journal.pone.0094895

**Published:** 2014-04-15

**Authors:** Tomohiro Yokota, Ryosuke Shiraishi, Takashi Aida, Kenji Iwai, Norika Mengchia Liu, Utako Yokoyama, Susumu Minamisawa

**Affiliations:** 1 Department of Life Science and Medical Bioscience, Waseda University Graduate School of Advanced Science and Engineering, Tokyo, Japan; 2 Department of Cell Physiology, Jikei University School of Medicine, Tokyo, Japan; 3 Cardiovascular Research Institute, Yokohama City University Graduate School of Medicine, Kanagawa, Japan; University of Iowa, United States of America

## Abstract

Ductus arteriosus (DA) closure follows constriction and remodeling of the entire vessel wall. Patent ductus arteriosus occurs when the DA does not close after birth, and this condition is currently treated using cyclooxygenase inhibitors. However, the efficacy of cyclooxygenase inhibitors is often limited. Our previous study demonstrated that low-dose thromboxane A_2_ receptor (TP) stimulation constricted the DA with minimal adverse effects in rat neonates. However, its effect on DA remodeling remains unknown. In this study, we focused on the impact of the exogenous TP stimulation on the DA remodeling, especially intimal thickening. Using DA explants from rat fetuses at embryonic day 19 as a *ex vivo* model and primary cultured rat DA smooth muscle cells from embryonic day 21 as a *in vitro* model, we evaluated the effect of TP stimulation on the DA remodeling. The selective TP agonists U46619 and I-BOP promoted neointima formation in the *ex vivo* DA explants, and TP stimulation increased DA SMC migration in a dose-dependent manner. Both effects were inhibited by the selective TP antagonist SQ29548 or the siRNA against TP. TP stimulation also increased DA SMC proliferation in the presence of 10% fetal bovine serum. LC/MS/MS analysis revealed that TP stimulation increased secretion of several extracellular matrix proteins that may contribute to an increase in neointima formation. In conclusion, we uncovered that exogenous administration of TP agonist promotes neointima formation through the induction of migration and proliferation of DA SMC, which could contribute to DA closure and also to its vasoconstrictive action.

## Introduction

The ductus arteriosus (DA) normally starts to close immediately after birth. However, it sometimes remains patent after birth; this condition is called patent DA (PDA). PDA occurs frequently in premature infants, and 60% to 70% of premature infants <28 weeks gestation receive medical or surgical therapy for PDA [1], [2]. The presence of PDA is more serious in premature infants than in full-term infant since premature infants with PDA are more likely to develop problems such as intraventricular hemorrhage, necrotizing enterocolitis, bronchopulmonary dysplasia, and congestive heart failure. PDA of full-term infants is usually associated with an inherent or genetic disorder and a structural abnormality of the DA, whereas PDA of the premature infants is related to prematurity of the DA itself. Although cyclooxygenase (COX) inhibitors such as indomethacin and ibuprofen have been widely used for prophylactic or symptomatic PDA treatment, they fail to close the DA in 20% to 40% of these premature infants [3], [4]. The high COX inhibitor failure rate currently leaves the clinician with surgical ligation as the only option. Although clinical studies showed that surgical ligation is a safe procedure [5]–[7], it has been reported that neurosensory impairment, bronchopulmonary dysplasia, and severe retinopathy are common adverse events after this surgery [8]. Therefore, an alternative pharmacological strategy to treat PDA is required.

Both functional and anatomical closures in sequence are necessary for permanent DA occlusion. Although functional closure is a relatively transient response and occurs as vasoconstriction after birth, anatomical closure refers to the constitutive developmental process of vascular remodeling during the perinatal period. Progressive intimal thickening that appears prominently during late gestation represents DA structural remodeling. This physiological intimal thickening is characterized by detachment of the endothelium from the internal elastic lamina (IEL), fragmentation of the IEL and loss of elastic fibers in the medial layer, deposition of extracellular matrix in subendothelial area, and migration of smooth muscle cells (SMC) into the subendothelial space [9], [10]. After these series of events, apoptosis and cytolytic necrosis are occurred and eventually forms a DA ligament [11], [12]. Immature or impaired vascular remodeling is often observed in human PDA patients and PDA animal models [11], [13]–[15]; it is difficult for the DA to close without the vascular remodeling taking place. Therefore, the effect of a newly developed therapy for PDA must take into account both functional and anatomical closure.

We previously demonstrated that low-dose thromboxane A_2_ (TXA_2_) receptor (TP) stimulation promotes functional closure of the rat DA with minimal adverse effects [16]. TXA_2_ is a lipid mediator that exhibits diverse physiological and pathological effects on the cardiovascular system; TXA_2_ is a strong vasoconstrictor that is involved in pathogenesis of vascular diseases including thrombosis, atherogenesis, and neovascularization [17]. This lipid mediator is synthesized from arachidonic acid using the COX pathway, via the pivotal intermediate prostaglandin H_2_ (PGH_2_), which in turn is converted to TXA_2_ by thromboxane synthase [17]. TP, a G-protein–coupled receptor that is expressed in many cell and tissue types [17], is known to be highly expressed in atherosclerosis lesions and involved in proliferation and migration of many cell types, including DA SMCs [18]–[20]. Moreover, TP activation plays an important role in accelerating atherosclerosis [21], [22]. However, the role of TXA_2_-TP in DA remodeling has not yet been investigated. Since several previous studies including ours have indicated that, in many species, exogenous TP stimulation functionally promotes DA closure through vascular constriction of the DA [16], [23], [24], we hypothesized that exogenous TP stimulation may play a role in DA remodeling.

## Methods

### Animals

Timed-pregnant Wistar rats were purchased from Japan SLC, Inc (Shizuoka, Japan). The DA was obtained from rat fetuses on gestational day 19 (preterm) and on gestational day 21 (term). The DAs on gestational day 19 remained immature physiological and morphological characters, whereas they were mature on gestational day 21 [3] All animals were cared for in compliance with the guiding principles of the American Physiologic Society. The experiments were approved by the Ethical Committees on Animal Experiments of Waseda University.

### Reagents

U46619 and I-BOP, selective TP agonists, and SQ29548, a selective TP antagonist, were purchased from Cayman Chemical (Ann Arbor, MI, USA). Prostaglandin E_2_ (PGE_2_), elastase type II-A, trypsin inhibitor type I-S, bovine serum albumin V, penicillin-streptomycin solution, DMEM, and HBSS were purchased from Sigma-Aldrich (Tokyo, Japan). Collagenase II was purchased from the Worthington Biochemical Corp. (Lakewood, NJ, U.S.A.). Collagenase/dispase was purchased from Roche Diagnostics (Tokyo, Japan). Fetal bovine serum was purchased from Invitrogen (Tokyo, Japan), and 3% buffered formalin was purchased from Wako Pure Chemical Industries, Ltd. (Osaka, Japan).

### Organ Culture

To study the *ex vivo* effect of TP stimulation on the premature rat DA, organ culture using DA explants was conducted, as previously described [25]. Briefly, pregnant rats were anaesthetized with isoflurane and fetuses were removed by caesarean section. Fetuses were sacrificed by decapitation. Fetal arteries including the DA and the aortic arch arteries on gestational day 19 were removed from the thoracic cavity. Cut segments were then incubated with U46619 (10^−6^ M), I-BOP (10^−6^ M), or PGE_2_ (10^−6^ M) for 48 hours in DMEM containing 0.1% fetal bovine serum, at 37°C under 5% CO_2_ and 95% ambient mixed air. To determine the effect of the TP antagonist SQ29548, DA explants were pretreated with SQ29548 (10^−5^ M) for 2 hours before incubation with TP agonists. Cultured explants were then fixed in 3% buffered formalin and embedded in paraffin. The sectioned segments in the middle portion of the DA were analyzed histochemically. Intimal thickening was defined as (neointima area/media area) ×100%. The average of at least 3 sections was used as the value for each tissue.

### Rat DA SMC Primary Culture

Vascular SMCs in primary culture were obtained from Wistar rat embryo DAs on gestational day 21, as described previously [26]. Pregnant rats were anaesthetized with isoflurane and fetuses were removed by caesarean section. Fetuses were sacrificed by decapitation. Confluent cells after 4 to 6 passages were used in the experiments. We confirmed that >99% of cells were positive for α-smooth muscle actin and showed the typical “hill-and-valley” morphology [27].

### SMC Migration Assay

The migration assay was performed using 24-well transwell culture inserts with polycarbonate membranes (8 µm pores; Corning Inc., Acton, MA, U.S.A.), as described previously with a few minor modifications [25]. Using a Boyden chamber fibronectin-coated membrane, we examined an acute (∼4 hours) effect of TP stimulation on SMC migration or TP inhibition with TP siRNA. To confirm the effect of TP antagonists, we treated SQ29548 for 30 minutes before TP stimulation. To examine the effect of TP stimulation on actively migrating SMCs, 10% FBS was used to stimulate SMC migration.

### Transfection of TP siRNA into DA SMCs

The antisense siRNA sequences targeting TP were 5′-CCUUGCUGCAGACGCUACCUGUCAU-3′ (siTP#1) and 5′-CCACGGAGCGCCAACUGCUCAUCUA-3′ (siTP#2) (Invitrogen). AllStars Negative Control siRNA (QIAGEN, Netherland) was used as a control non-silencing siRNA. DA SMCs were serum deprived and transfected with siRNA (80 pmol) using the RNAi max transfection reagent (Invitrogen), according to the manufacturer's instructions. Cells were then incubated in 0.1% FBS containing DMEM for an additional 24 hours before the start of each experiment.

### Fluorescence-activated Cell Sorting (FACS) analysis

Pregnant rats were anaesthetized with isoflurane and fetuses were removed by caesarean section. Fetuses were sacrificed by decapitation. Pooled tissues from the DA or the aorta were obtained from three litters of timed-pregnant Wistar rats, which was approximately thirty fetuses. Tissues were treated with collagenase-dispase enzyme mixture as described previously [26]. Approximately 1.0×10^6^ cells were obtained from combined whole DA tissues from the three litters. These cells were incubated with FITC-conjugated anti-CD31 and APC/Cy7-conjugated anti-CD45 antibodies as cell surface markers for EC and hematopoietic derivation cells, respectively as described previously [28]. Briefly, in order to confirm the nonspecific binding of antibodies to cells, we also prepared cells incubated with a fluorescence conjugated anti-control IgG antibody. When dead cells were incubated with PI solutions (Dojindo, Kumamoto, Japan), approximately 30% of the cells (3.0×10^5^ cells) were stained by the PI solutions, indicating that they had died during isolation. The dead cells were then removed from further analysis. All cells were detected and sorted using a BD FACSAria^TM^II (Becton Dickinson, San Jose, CA, USA). In order to obtain as many cells as possible without affecting their purity, we set the FACS's sorting mode on “purity”, which allowed the machine to automatically remove any possible forms of contamination, such as doublet cells, yielding a purity greater than 99%. The sorted cells were placed into a 1.5 ml centrifuge tube containing 500 µl DPBS (Wako) and 0.4 µl RNase inhibitor (Roche, Meylan, France).

### Quantitative Reverse Transcriptase (RT) – PCR Analysis

Total RNA isolation from pooled tissues or cultured SMCs, cDNA generation and RT-PCR analysis for TP were performed as described previously [27]. Total RNA was isolated from cultured DA SMC from Wistar rat embryos, cDNA generation and RT-PCR analysis were performed as described previously [29]. A Taqman Gene Expression Assay (Rn_00690601_m1) (Applied Biosystems, Foster City, CA, USA) was used for TP as described previously [16].The abundance of each gene was determined relative to an internal control using 18 s ribosomal RNA. For each RT-PCR experiment, which included a RT negative control, we confirmed that there was no amplification in any reaction.

### BrdU uptake assay

Cell proliferation was measured using a chemiluminescent immunoassay, based on the amount of BrdU incorporation during DNA synthesis (Cell Proliferation ELISA, Roche, Tokyo, Japan). SMCs were reseeded into a 96-well culture plate at an initial density of 2×10^4^ cells per well. These cells were starved in 0.5% FBS containing DMEM for 24 hours and then incubated with U46619 (10^−6^ M) in serum-free DMEM or 10% FBS containing DMEM, or 10% FBS containing DMEM for 24 hours in DMEM with humidified 5% CO_2_ and 95% ambient mixed air at 37°C. Cells underwent BrdU labeling for 2 hours, according to the manufacturer's instructions.

### Hyaluronan Quantification

The amount of hyaluronan in the cell culture supernatant was measured as previously described [26].

### Liquid chromatography mass spectrometry (LC/MS/MS) analysis of TP-induced protein secretion from DA SMCs

Cultured DA SMCs were passaged 5 times and seeded on a 6-cm culture dish at a concentration of 5×10^4^ cells/mL. Cells were starved using serum-free DMEM for 24 hours. These cells were then stimulated with a concentration of 10^–6^ M U46619 for 48 hours. DA SMC culture supernatant was collected after U46619 (10^−6^ M) stimulation. Reduced buffer (500 µL) contained 8 M Urea (Sigma-Aldrich), 500 mM Tris-HCl (Sigma-Aldrich; pH 8.5) and 2.5 mM EDTA (Dojindo, Tokyo, Japan) were added and ultrafiltration using Amicon Ultra Centrifugal Filters (Millipore, Billerica, Massachusetts, USA) was performed twice. After preparing 10 µg of proteins, 15 µL of reduced buffer and 5 µL of 40 mM DTT (Sigma-Aldrich) were added and incubated for 1.5 hours at 37°C. Then, 5 µL of 250 mM iodoacetamide (Wako) was added and incubated for 30 minutes at room temperature under dark conditions. After adding 180 µL of 50 mM ammonium hydrogen carbonate (Sigma-Aldrich), proteins were treated with Promega Sequencing Grade Modified Trypsin (Promega, Tokyo, Japan), and 0.08 µL of 0.1 M calcium chloride (Nacalai Tesque, Kyoto, Japan) was then added and incubated for 16 hours at 37°C. After incubation, 1 mL of solution A (0.1% formic acid:acetonitrile  = 98∶2), 1 mL of solution B (0.1% formic acid:acetonitrile  = 2∶98), 100 µL of eluate 1 (solution A:solution B = 60∶40) and 100 µL of eluate 2 (solution A:solution B = 30∶70) were prepared. The pipetting cycle (5 times for solution B, 5 times for solution A, 10 times for the sample solution, 5 times for solution A, 10 times for eluate 1, 10 times for eluate 2) was performed 3 times using Monotip C18 (GL Sciences, Torrance, CA, USA). The mixture of eluate 1 and eluate 2 was dried under reduced pressure, and 5 µL of a mixture of 20% acetonitrile (Kanto Chemistry, Tokyo, Japan) and 0.02% formic acid (Wako) were added. After mixing, 0.02% formic acid was added and mixed. Peptide fragments (1 µg) were analyzed using liquid chromatography and separated using a gradient for 180 minutes. The HPLC column (GL Sciences) was washed using a gradient with solution B for 180 minutes. Separated peptide fragments were analyzed using a NanoFrontier eLD (Hitachi, Tokyo, Japan). A mascot server was used to obtain a list of the detected proteins, and comparative protein quantification between samples with TP stimulation and no stimulation was performed using Mass Navigator software (Mitsui Knowledge Industry, Tokyo, Japan). Proteins that have high-matching score were selected at first, and then we chose the candidate proteins showing that the ratio of TP stimulation to non-stimulation was higher than 1.4 and less than 0.6.

### Statistical Analysis

All data are presented as the mean ± SEM. The Student's unpaired *t* test was used to compare mRNA expression in the SMCs and ECs. Comparisons between data from multiple groups were performed using an unpaired ANOVA followed by the Student's-Newmann-Keuls test. *P* values <0.05 were considered statistically significant.

## Results

### TP agonists promoted intimal thickening in the DA explants

**Figure 1:**
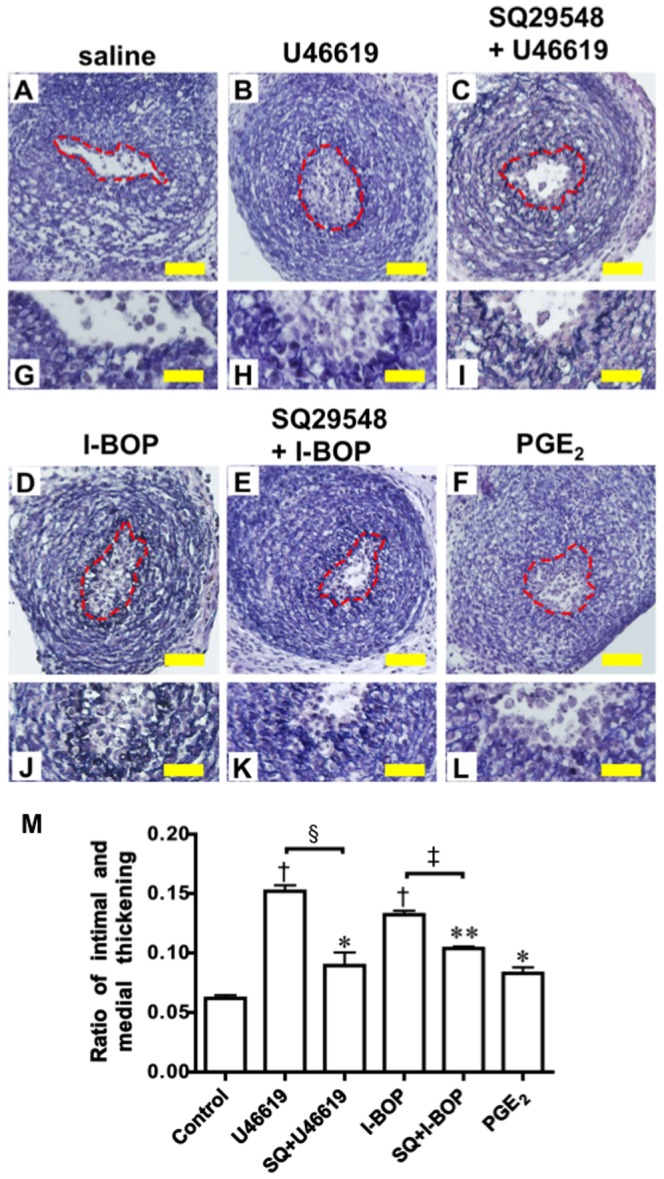
TP stimulation induced DA intimal thickening. (A–F) DA explants incubated with saline- (A), U46619- (B), SQ29548+U46619- (C), I-BOP- (D), SQ29548+I-BOP- (E), or PGE_2_- (F) containing medium for 48 hours. Broken red line indicates the elastic lamina. (G–L) Magnifications of A to F. (M) Evaluation of the intimal thickening. Values are expressed as the mean ± SEM. n = 3 to 7. *, **, and ^†^ indicate *p*<0.05, *p*<0.01, and *p*<0.001 versus control, respectively; ^§^indicates *p*<0.001 versus U46619; ^‡^indicates *p*<0.001 versus I-BOP. Scale bar: 40 µm (A–F) and 20 µm (G–L).

We performed an organ culture assay to determine the effect of TP agonists on intimal thickening using rat DA explants from embryonic day 19, at which the endogenous intimal thickening is not visible and the DA shows less constrictive response than mature DA. Intimal thickening was fully developed in the DA explants after immature rat DA was exposed to TP agonists for 48 hours in culture (**Figure 1B, D**). The intimal thickening was 2.9-fold or 2.4-fold greater in the presence of the TP agonists U46619 (10^−6^ M; n = 5) and I-BOP (10^−6^ M; n = 4), respectively, compared to control saline (n = 4) (**Figure 1M**). The increase in response to TP agonists was greater than PGE_2_-induced intimal thickening (10^−6^ M; n = 7). The TP antagonist SQ29548 (10^−5^ M) significantly inhibited the U46619- and IBOP-mediated intimal thickening by 0.3-fold or 0.31-fold (n = 4 or n = 3), respectively (**Figure 1C, E, M**). These data indicated that TP stimulation promoted intimal thickening in the DA explants.

### TP mRNA is highly expressed in DA smooth muscle cells compared to DA endothelial cells

**Figure 2:**
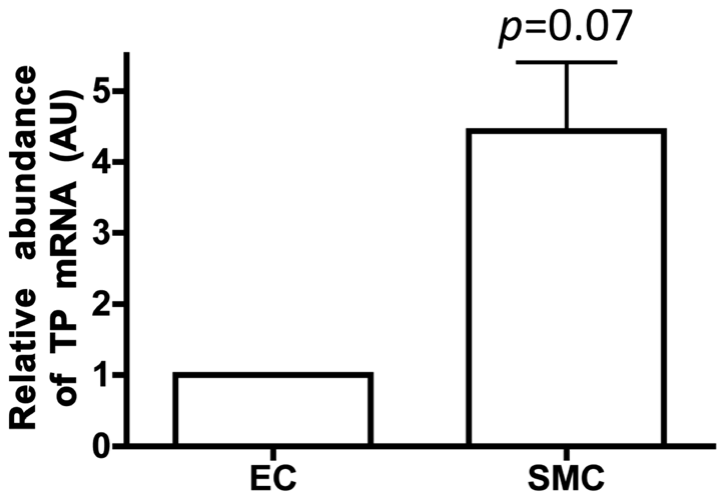
TP expression was higher in smooth muscle cells than in endothelial cells. Relative abundance of TP mRNA in DA endothelial cell (EC) and smooth muscle cell (SMC) on gestational day 21. Values are expressed as the mean ± SEM. n = 3.

The DA mostly consists of SMCs and endothelial cells (ECs). To determine the expression levels of TP mRNA in these two cell types, we quantified the TP mRNA expression in SMCs and ECs isolated using FACS. The TP mRNA expression level was higher in SMCs than in ECs (**Figure 2**). Our data is consistent with the previous report that demonstrated the expression of TP in human DA [18]. Taken together with these results and our ex vivo data, DA SMCs mainly contribute to TP-induced neointimal formation in the DA.

### TP stimulation promotes DA SMC migration

**Figure 3:**
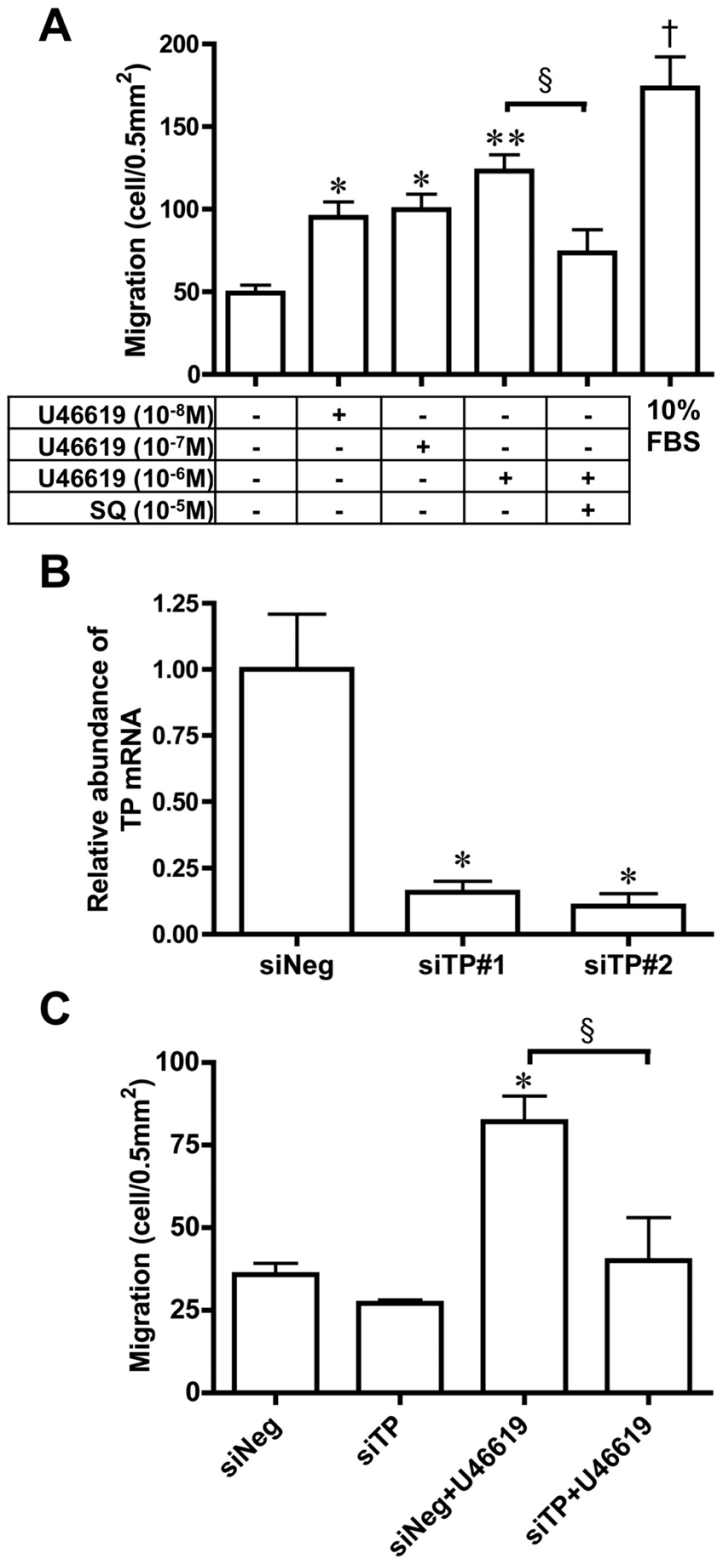
TP stimulation increased DA SMC migration. A) The effect of the U46619 on DA SMCs' ability to migrate. Cells were incubated with a migration stimulator for 4 hours. Values are expressed as the mean ± SEM. n = 4 to 6.*, **, and ^†^ indicate *p*<0.05, *p*<0.01, and *p*<0.001 versus control, respectively; ^§^ indicates *p*<0.05 versus U46619 10^−6^ M. (B) TP mRNA expression in DA SMC treated with siControl, siTP#1, or siTP#2. Values are expressed as the mean ± SEM. n = 3. * indicates *p*<0.01 versus siNeg. (C) The effect of siRNA-induced TP knockdown on U46619-induced DA SMC migration. Cells were transfected with siControl or siTP#2 before treatment with U46619. Transfected cells were incubated with/without U46619-containing media for 4 hours. Values are expressed as the mean ± SEM. n = 3. *indicates *p*<0.01 versus siNeg; ^§^ indicates *p*<0.01 versus siNeg+U46619.

SMC migration from the vascular media into the endothelial layer is an important vascular remodeling process during intimal cushion formation [30], [31]. U46619 induced DA SMC migration in primary culture (n = 10) in a dose dependent manner (**Figure 3A**), which was inhibited by SQ29548. In addition, we found that TP siRNA transfection significantly inhibited TP mRNA expression (∼90% decrease, n = 3) and the TP-induced migration (53% decrease, n = 3) (**Figure 3B, C**). TP knockdown by its siRNA didn't affect the endogenous activity of the DA SMC migration.

### U46619 promotes DA SMC proliferation in the presence of FBS

**Figure 4:**
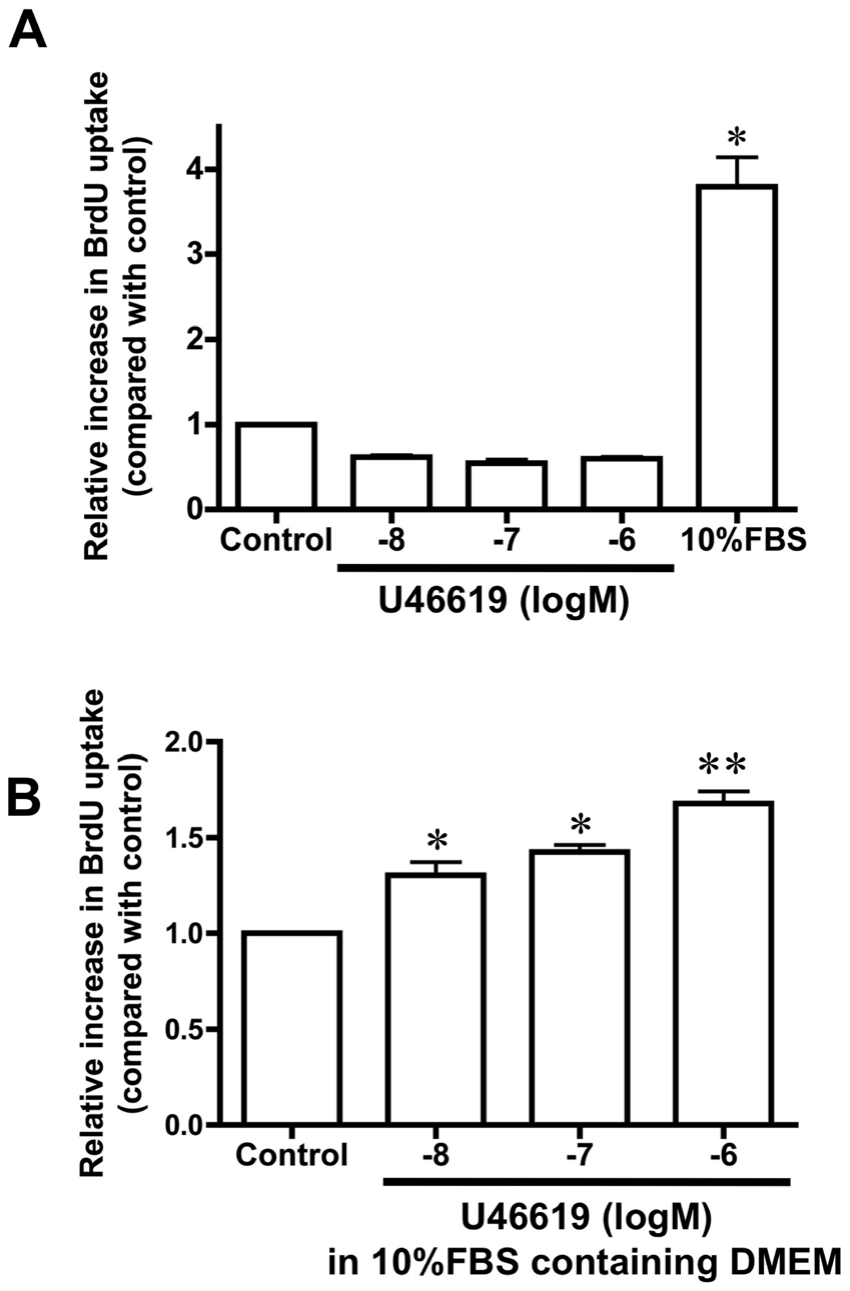
TP stimulation increased the DA SMC proliferative properties. TP stimulation caused DA SMC proliferation in the presence of FBS DMEM but not in the absence of FBS. (A) In the absence of FBS, the TP agonist did not seem to promote DA SMC proliferation. (B) In the presence of FBS, TP stimulation induced DA SMCs proliferation in a dose-dependent manner. Values are expressed as the mean ± SEM. n = 3∼4. * and **indicate *p*<0.01 and *p*<0.001 versus control, respectively.

Since TP stimulation induced cell proliferation in some arteries and induced proliferation involved in DA remodeling [17], [19], we examined whether U46619 promoted DA SMC proliferation. Using the BrdU uptake assay, we found that U46619 did not promote DA SMC proliferation in the absence of FBS (**Figure 4A**; n = 4). However, in the presence of 10% FBS, U46619 promoted DA SMC proliferation in a dose-dependent manner (**Figure 4B**; n = 3). In the in vivo environment, migrating DA SMCs are exposed to blood that contains many different growth factors. Therefore, it is not surprising that TP-stimulation activates DA SMC proliferation only in the presence of serum.

### TP stimulation promoted the secretion of several DA SMC extracellular matrix proteins

**Figure 5:**
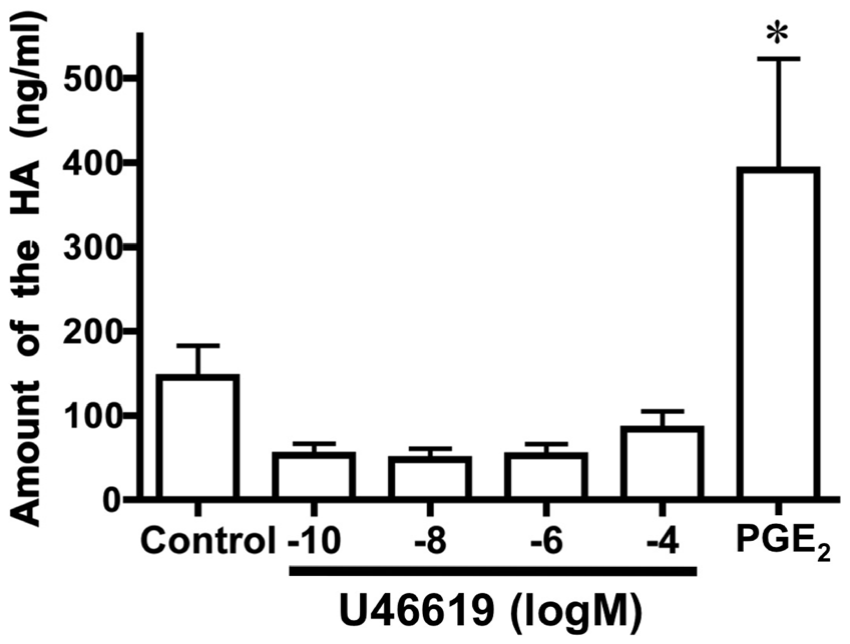
TP stimulation did not promote hyaluronan production. The amount of hyaluronan (HA) in the supernatant from the cultured DA SMCs. Values are expressed as the mean ± SEM. n = 4. * indicates *p*<0.01 versus control.

Because PGE_2_ is known to promote intimal DA thickening through an increase in hyarulonan [25], we quantified hyaluronan production in the supernatant from cultured DA SMCs. We found that U46619 did not increase hyaluronan production (**Figure 5**), indicating TP-induced intimal thickening is independent to PGE_2_-induced intimal thickening. As to the mechanistic insight of TP-induced intimal thickening including migration and proliferation, it's unclear. Therefore, to examine secreted factors and extra cellular matrices that promote migration and proliferation of the DA SMCs, we used LC/MS/MS to analyze the supernatant from cultured DA SMCs in the presence or absence of U46619. TP stimulation increased the secretion of biglycan, protein nephroblastoma overexpressed homolog (NOV/CCN3), transgelin, connective tissue growth factor (CTGF/CCN2), actin, and fibronectin, and decreased that of elastin and insulin-like growth factor-binding protein 2 (**Table 1**). Several of these proteins are known to be involved in cell proliferation and migration [32], [33], [34]. Especially, we found that TP stimulated the secretion of fibronectin, known to be abundant in the DA and involved in DA remodeling [30], [35].

**Table 1:** TP-induced secreted protein from the DA smooth muscle cells.

Protein Name	TPago/serum-free (Fold change±SEM)
Biglycan	2.58±1.49
Protein NOV homolog	1.98±0.35
Transgelin	7.69±3.81
Connective tissue growth factor	5.88±3.77
Actin, cytoplasmic 2	3.46±1.34
Fibronectin	1.49±0.16
Elastin	0.45±0.09
Insulin-like growth factor-binding protein 2	0.51±0.06

## Discussion

The present study demonstrated that exogenous TP stimulation promoted neointima formation in the rat DA explants probably because of the induction of SMC migration, proliferation, and secretion of ECM proteins. It should be noted that the PDA phenotype has not been reported in TP knockout mice [36] and that a TP antagonist itself does not inhibit DA closure after birth [16]. Therefore, it is unlikely that intrinsic TXA_2_ -TP signal plays a pivotal role in spontaneous DA closure.

Our previous study demonstrated that the PGE_2_-EP4 signal promotes DA closure through hyaluronan-mediated neointima formation [25], whereas the present results showed that hyaluronan did not involve TP stimulation-induced neointima formation. The TP signaling pathway is capable of stimulating proliferation in several kinds of SMCs, including coronary, bronchial, and aortic arteries. In addition, several studies have reported a relationship between TP activation and the development of atherosclerosis [22], [37]. Our results suggested that TP stimulation induced DA SMC proliferation in the presence of serum. Gao et al. demonstrated that TP stimulation-induced PKC enhances TP-Gi coupling and the Gβγ released from activated Gi proteins promotes the p44/42 mitogen-activated protein kinase (p44/42 MAPK)/extracellular signal-regulated kinase (ERK) through Src, which is the epidermal growth factor receptor (EGFR)-dependent pathway [38]. Since TP/MAPK/ERK signaling is active in several vascular SMCs, TP-EGFR-coupled stimulation might induce DA SMC proliferation in FBS that contains EGF. Our results also suggested that TP stimulation induces DA SMC migration. Yun et al. determined that TP induced mesenchymal stem cell migration through ERK and p38 MAPK activation [39]. Vascular SMC proliferative activity is known to be regulated by the ERK, p38, and JNK pathways [40], [41]. Our results together with these reports suggest that TP/ERK and/or TP/p38 MAPK signaling might be involved in DA SMC migration.

Research on the development of DA intimal thickening showed that this process is similar to what has been described for atherosclerosis [42], [43]. Since TP is involved in the development of atherosclerosis [37], it is highly possible that TP stimulation induces remodeling of the DA. In DA remodeling, it is known that ECMs, such as glycosaminoglycans, fibronectin, laminin, and collagen, are involved in this process [44]. Our previous report showed that EP4/PGE_2_ signaling plays a critical role in DA remodeling through a change in hyaluronan production [25]. However, our data showed that TP stimulation did not induce hyaluronan production in DA SMCs. Instead, there are several TP-induced proteins secreted that may be candidate ECMs and that are involved in TP-induced DA remodeling. Biglycan, one of the candidate ECMs known to be involved in pro-inflammatory events such as atherosclerosis, induced a more migratory phenotype by altering the cell shape and increasing the expression of several effectors genes that are involved in cell migration [33], [34]. In our previous publication conducting DNA microarray analysis from DA at preterm, term, and postnatal period, the expression of biglycan remains higher at term and early postnatal period than that at preterm [45]. Since DA intimal thickening is initiated from term period, it could be possible that biglycan is associated with initiation and/or promotion of intimal thickening. In addition, this ECM has a modified form, specifically the form with hyperelongated glycosaminoglycan chains, which shows enhanced lipid binding *in vitro* and increases lipid deposition in the vessel wall in animal models of atherosclerosis [33], [46]. Considering the relationship between biglycan and glycosaminoglycan, it is probable that TP-induced up-regulation of biglycan could be involved in TP-induced remodeling. In addition, biglycan is known to increase collagen and fibronectin expression in human airway SMCs, and fibronectin is known to promote DA SMC migration [47], [48]. Moreover, our data shows that TP induced an increase in fibronectin production. Therefore, biglycan could be an interesting target to uncover the mechanism on TP-mediated induction of DA remodeling.

Surgical ligation is performed to close the PDA when treatment with COX inhibitors is contraindicated or failed [49]. Although the morbidity and mortality rates for surgical ligation are low, neurosensory impairment, bronchopulmonary dysplasia, and severe retinopathy have been reported after surgery [8]. We previously showed that low-dose TP stimulation of neonatal rats constricted the DA, but did not cause constriction in vessels such as the marginal arteries of the colon, a decrease in blood flow in the tail, or microthrombosis in the pulmonary capillary arteries [16]. In addition, we have not observed a decrease in cardiac output or sudden death in the neonatal rat in the short term. Taken together with our current results, low-dose TP stimulation induced both DA constriction and remodeling and had minimal adverse effects. TP agonists are not currently approved for clinical use and more time and effort is needed to obtain approval for this indication, because continuous stimulation by TP agonists are reported to cause adverse effects, such as neonatal pulmonary hypertension and nephrotic disease of kidney, in animal models [17], [50]. Although our previous report demonstrated that TP stimulation did not induce the constriction of the pulmonary artery and the aorta in the *in vivo* premature rat fetuses [16], the application of TP agonists for premature infants should be very cautious because they are intrinsically fragile in their hemodynamics. Therefore, a detailed safety evaluation of low-dose TP stimulation to assess possible long-term adverse effects in larger animals should be required before any clinical applications. Here we propose that low-dose TP agonists may serve as a possible pharmacological therapeutic strategy for DA closure, although further investigation is required to support this indication.
